# Bee Pollen Role in Red Winemaking: Volatile Compounds and Sensory Characteristics of Tintilla de Rota Warm Climate Red Wines

**DOI:** 10.3390/foods9080981

**Published:** 2020-07-23

**Authors:** Antonio Amores-Arrocha, Pau Sancho-Galán, Ana Jiménez-Cantizano, Víctor Palacios

**Affiliations:** Department of Chemical Engineering and Food Technology, Vegetal Production Area, Faculty of Sciences, University of Cadiz, Agrifood Campus of International Excellence (ceiA3), IVAGRO, P.O. Box 40, 11510 Puerto Real, Cadiz, Spain; pau.sancho@uca.es (P.S.-G.); ana.jimenezcantizano@uca.es (A.J.-C.); victor.palacios@uca.es (V.P.)

**Keywords:** bee pollen, Tintilla de Rota, alcoholic fermentation, warm climate, volatile compounds, sensory profile, fermentative activator, red winemaking, red wines

## Abstract

One of the main aspects that define wine quality is its aromatic profile. Nutritional deficiencies in musts can lead to olfactory defects and a decline in quality. Commercial activators and nutrients are usually added to the must in these cases. The natural composition of bee pollen can provide all the necessary nutrients for yeasts. This investigation aims to analyze the impact of pollen addition on the profile of volatile and sensory compounds in Tintilla de Rota warm climate red wines. Volatile compounds were measured by Gas Chromatography–Mass Spectrometry, Odorant Activity Values analysis to find out each compound’s fragrant participation, and sensorial analysis was conducted for a qualified panel of wine-tasters. As a result of the chromatographic analysis, 80 volatile compounds of different chemical families were identified and quantified. Bee pollen increased mainly isoamyl alcohol, esters, and terpenes compounds families in wines. Odorant Activity Values analysis showed an increase in fruity odorant series mainly, followed by floral, for all wines with pollen addition. The sensory analysis showed that low pollen doses (0.1 g/L and 0.25 g/L) increased tasting notes of fruit and floral attributes and fruit and floral odorant series as well, highlighting an increase in red and black fruit notes mainly. On the other hand, high doses deviated the sensory profile towards fleshy stone fruit, and raisin fruit, mostly. In addition, high bee pollen doses produce an increase in the odorant category responsible for the chemical, fatty, and grassy aromas mainly, and high and intermediate dose (1 g/L) an increase in the earthy notes in the aromas. Therefore, low bee pollen doses (0.1 and 0.25 g/L) can improve both the aromatic compound profile, as well as the Odorant Activity Values levels and the sensory profile in Tintilla de Rota red wines.

## 1. Introduction

The present tendency in wine consumption focuses on well-structured wines and full bodied in the mouth [1]. In addition, it is remarkable that the aroma of wine constitutes an important factor in consumer preference [2]. The compounds involved in the aroma can be derived from many sources: alcoholic fermentation, from biosynthesis, and from the conversion from neutral grape compounds to active components [3]. The majority of esters, as well as higher alcohols, volatile acids, and compounds within the thiol and terpene families, which are varietal compounds, are produced during alcoholic fermentation by yeast [4].

A complex and varied nutrient composition rich in amino acids, as well as fatty acids and vitamins in grape musts [5,6] ensure an adequate alcoholic fermentation development. In order to obtain them, weather conditions should be suitable during the grapes ripening stage [7]. Unfortunately, the current context of global warming is giving rise to problematic ripening processes directly responsible of changes in grape must composition [8,9], generating nutrient deficiencies for the yeasts. In this regard, potential difficulties may appear during alcoholic fermentation and, as a consequence, sensory profile defects in wines [10]. In warm climate areas, such as Southern Andalusia (Spain), wines may experience a loss in aromatic and sensory expression [11]. In order to confront these new environmental conditions in traditional winegrowing regions, several authors suggest cultivating autochthonous varieties better adapted to them. However, currently the main solution used by winemakers to resolve these problems is to employ commercial synthetic fermentative activators and nutrients [12,13].

Bee pollen is a natural product with a rich composition mainly composed by proteins, vitamins, minerals, and carbohydrates, but also of amino acids, fatty acids, sterols, phospholipids, carotenoids and polyphenols [14,15,16,17,18,19]. Amores-Arrocha et al. [20,21] stated bee pollen as a “Green nutrient activator”, as they observed improvements in fermentation kinetics (increased fermentation rate, reduction of the yeast lag phase and increased cell multiplication), both in white and red winemaking processes, although its use is currently not legally authorized for industrial processing. In addition, at low doses, bee pollen has not affected the red wine’s physicochemical composition or color parameters [20] Other published studies showed how bee pollen use improved both volatile and sensory compounds profiles of young white wines [22] as well as the aging kinetics and sensory profile of white wines undergoing biological aging [23]. Therefore, the aim of this research is to explore the effect of bee pollen use on the profile of both volatile compounds and sensory profile in red wines elaborated with an autochthonous grape variety: Tintilla de Rota.

## 2. Materials and Methods

### 2.1. Experimental Layout

Tintilla de Rota clusters of grapes were collected from vineyards of the privately-owned winery Luis Pérez, located in Jerez de la Frontera (southern Andalusia, Spain) (36°42′00.6′′ N, 6°11′34.0′′ W, 100 m above sea level). Vineyard soil was mainly limestone, known locally as “albariza”. Grapes were destemmed and crushed. To avoid oxidations, the skins and grape musts mixture was sulphited with 25 mg/kg of K_2_O_5_S_2_ (Sigma-Aldrich Chemical S.A., Madrid, Spain), tempered (20 °C) and no pH correction. A control (0 g/L) and six different doses (0.1, 0.25, 1, 5, 10 and 20 g/L) of commercial grounded bee pollen (Valencia, Spain) were studied. Bee pollen was added to each fermenter and homogenized with the paste, before adding the yeast inoculum. All vinifications assays were carried out in duplicate using temperature controlled glass fermenters (V = 5 L). Commercial yeast, *Saccharomyces cerevisiae* Lalvin 71B^®^ strain (Lallemand, Barcelona, Spain), 10 g/hL inoculum was used to perform the alcoholic fermentation (AF). For malolactic fermentation (MLF), once AF was over, a Lactic Acid Bacteria (LAB) inoculum (1 g/hL) *Oenococcus oeni* S11B P2 Instant (Laffort, Bordeaux, France) was added.

### 2.2. Analysis of Volatile Compounds and Odor Activity Values (OAV)

Volatile compounds and their corresponding OAVs were analyzed following the methodology indicated by several authors [22,24]. Odor series were assigned to each component based on the main odor descriptor according to Peinado et al. [25], to obtain quantitative information from chemical analysis based on target criteria.

### 2.3. Sensory Wines Evaluation

A 10-member panel of trained and instructed experts experienced in wine tasting performed the evaluations. The sensory analysis was carried out in individual cabins equipped with a lighting control system. 50 mL of wine were provided to each taster in a regular wine-tasting glasses (ISO 3591, 1997) [26], topped with glass to avoid volatile compounds evaporation. Each sample was encoded using a random three-digit code to be tasted according to the order indicated. The wines were then served at a temperature of 20 ± 2 °C. In order not to overload judges, each of them performed the same tasting on a two different days in order to carry out the sensory analysis more than once presenting wine glasses randomly each time. Specific tasting notes were given to each taster and each attribute was scored according to an increase in intensity, based on a 10-point rating scale. All the sensorial characteristics employed in the tasting sheets have been selected taking into account the commercial wine style sensorial profile and following Jackson [27] tasting descriptors for red wines. Fruity, floral, and spicy aromas, as well as acidity, astringency, bitterness, sweetness, and milky notes were evaluated as generic attributes. In addition, the global judgment was added, as an attribute which encompasses the balance of all the attributes in conjunction with each other. Fruits groups (red, black, white, tropical, citrus, fruits with bone, raisin, and nuts), flowers groups (white, red, blue), vegetable, spices, woody, toast, balsamic, minerals, animals, and chemical, were the specific attributes evaluated by the judges.

### 2.4. Data Treatment

Means and standard deviations with significant differences were determined by bidirectional ANOVA and Bonferroni’s multiple range (BSD) test; *p* < 0.05 was considered significant (GraphPad Prism version 6.01 for Windows, GraphPad Software, San Diego, CA, USA). For statistical significance, all tests were conducted in triplicate (*n* = 3). Principal Components Analysis (PCA) was performed using the SPSS 24.0 statistical computer package (SPSS Inc., Chicago, IL, USA).

## 3. Results and Discussion

### 3.1. Evaluation of the Effects of the Addition of Pollen on Wine Volatile Compounds and their Corresponding Odorant Activity Values (OAV)

#### 3.1.1. Higher Alcohols and Methanol

Higher alcohols total concentrations were found in order of 200 mg (Table 1). In general, higher alcohols are not affected by the use of pollen, except for the 0.25 g/L dose, where isoamyl alcohol is slightly higher, without exceeding 400 mg/L. Taking into account the red wines, Yeast Assimilable Nitrogen (YAN) levels [20] were higher than those of white wines [21], it could be expected higher alcohol levels, however, this does not occur. These results imply that there is no relationship between YAN and increased alcohol production, and skin presence is buffering the effect of pollen.

#### 3.1.2. Aldehydes

In general, the aldehyde content of red wines (3066–5699 µg/L) was lower than white wines [22]. No correlation was observed between aldehyde formation and pollen dose, and their contents fluctuate between the different doses. As might be expected, acetaldehyde was the major compound in this family, followed by benzeneacetaldehyde. Both compounds contributed to the wines sensory profile with nutty and floral notes. Additionally, nonanal and 3-methyl-butanal were identified, which, due to low perception thresholds, contribute to the wines’ aromatic profile.

#### 3.1.3. Alcohols

Alcohol content was representing between 4–5.5% of the total volatile compounds (Table 1). Its values showed fluctuations in all samples, without following any correlation. This behavior is mainly marked by phenylethyl alcohol, which is the main alcohol, alongside with 1-pentanol and 1H-Indole-3-ethanol. Both phenylethyl alcohol and 1H-Indole-3-ethanol showed fluctuations while 1-pentanol together with most alcohols tended to increase significantly with respect to the control.

#### 3.1.4. Acids

Acid compounds content of red wines is much lower than that of white wines [22], which could be attributed to varietal character and also to skins presence during alcoholic fermentation. Some authors have found that grape skins provide fatty acids (oleic and linoleic), reducing the of volatile acids synthesis by yeasts [28,29]. However, analyzing the acid profile, two very different behaviors can be distinguished (Table 1). On the one hand, both control as well as low and intermediate doses (0.1–1 g/L) presented concentrations between 1688.54 and 2177.68 µg/L. On the other hand, the high doses (5–20 g/L) showed a greater range of concentration, with values between 3265.30 and 4064.52 µg/L. This could be due to autolysis phenomena produced between the end of AF and MLF, since volatile fatty acids from the cell membranes (hexanoic, octanoic, decanoic, and dodecanoic fatty acids) may be transferred into the medium via cell lysis [28,30].

#### 3.1.5. Esters

Esters family percentages of representation in red wines were observed between 10.86–22.34% (Table 1). Therefore, with the bee pollen addition, the formation of esters is favored in Tintilla de Rota red wines. A linear correlation is also observed between esters concentration and pollen doses (R^2^ = 0.96). In most cases, esters increase with the dose of pollen: ethyl acetate (R^2^ = 0.96), isoamyl acetate, ethyl octanoate, diethyl succinate (R^2^ = 0.68), phenethyl acetate, ethyl lactate (R^2^ = 0.64), methyl hexadecanoate, hexadecanoic acid, ethyl ester, ethyl 8-nonanoate (R^2^ = 0.90), hexyl acetate (R^2^ = 0.78), ethyl nonanoate (R^2^ = 0.71) and diethyl malate (R^2^ = 0.67). According to some authors, after MLF, an increase in esters concentration, ethyl acetate, ethyl hexanoate, ethyl lactate, and ethyl octanoate can be observed [31,32,33,34,35,36]. It should be noted that the main ester is ethyl acetate, whose descriptor is acrylic flavor, so it could be one of the compounds responsible for providing unpleasant aromas [37], especially for wines elaborated with the highest bee pollen doses (10 g/L and 20 g/L). This effect could be promoted because of YAN content and not because of pollen.

#### 3.1.6. C6-Alcohols

C6-alcohols were found in a range between 490 and 587 µg/L (Table 1). These compounds have fresh herbs and vegetables flavors and could be formed prior to AF via enzymatic action on their major precursors (linoleic acid and linolenic acid) [38]. During AF, these compounds are reduced to alcohols by yeast, mainly hexanol and hexenol, being the second most fragrant, but it was found in lower concentration in wines [38,39]. There are also these compounds present in grapes in their glycol form, but in lower concentration than in the pre-fermentation stages. Furthermore, the presence of antioxidant polyphenolic substances from the skins allows a low oxidation of final red wines.

#### 3.1.7. Terpenes

Terpenes represented very small percentages within the different volatile compound families of the obtained red wines (<0.1%) (Table 1). These compounds are typical of some grape varieties, and provide important floral notes in wine aroma [40]. The main terpenes able to contribute to wine aroma are naturally found in grape skins. In parallel to enzymatic actions, winemaking operations favors the extraction of these compounds from the grape must (macerations, pump-over, or head dipping) [39]. As can be seen in Table 1, most of the terpenes increased with pollen addition, with 8-hydroxylinalool standing out. However, there was no direct correlation between the applied dose and these family of compounds. It could be suggested that in addition to the grape skins, bee pollen was directly providing terpenes to wines.

#### 3.1.8. Phenols

Most of the phenolic compounds are formed during AF, by decarboxylation of hydroxycinnamic acids carried out by *Saccharomyces cerevisiae*. Phenols were found in very low concentrations in red wines. 4-vinylguaiacol and acetovainillone were detected, providing spicy notes as typical varietal aromas of Tintilla de Rota wines. Phenol contents fluctuated with pollen doses unable to establish any pattern in this regard.

#### 3.1.9. Thiols, Acetals, and Norisoprenoids

Thiols, acetals, and norisoprenoids represented the three minor compounds families of the volatile compound profile of red wines samples. Thiols were represented by 3-(methylthio)-1-propanol, whose concentration increased by bee pollen addition. This compounds formation has its origin in cysteine precursors present in grape must [39] which are degraded by yeasts to give rise to thiols. Thiols increase also could be justified by the natural richness of bee pollen in cysteine [41,42,43]. Acetals are compounds that can come from acetaldehyde and glycerol, however they are more commonly found at high levels in fortified wines [39,44]. Acetals family was represented by 1-(1-Ethoxyethoxy)-pentane, whose concentration showed a rising trend from the 5 g/L dose. Norisoprenoid family was represented by 3-oxo-α-ionol, whose concentration increased with pollen addition especially at high doses (10 and 20 g/L pollen). Some norisoprenoid, such as 3-oxo-α-ionol, have been found in several types of honey [45]. Considering bee pollen as the main raw material of honey, it is possible these compounds are being released by pollen during winemaking.

#### 3.1.10. Lactones

Certain lactones have fermentation origins and are able to take part in wine aroma [46]. Both compounds identified in red wines studied were dihydro-5-pentylglycol-2(3H)-furanone and 2.3-dihydro-benzofuranone, representing under 0.1% of the total amount of volatile compounds. Dihydro-5-pentyl-2-(3H)-furanone showed a fluctuating behavior, while 2.3-dihydro-benzofuranone remained constant at all doses.

#### 3.1.11. Principal Component Analysis (PCA) of Volatile Compounds 

Table 2 shows the results of the loadings of the factors extracted in the analysis of the Principal Components Analysis (PCA). Concentration of total volatile compounds by aromatic families: higher alcohols, methanol, acids, C6-alcohols, alcohols, phenols, terpenoids, esters, aldehydes, acetals, norisoprenoids, and lactones, were variables included. PCA analysis extracted three factors that represent more than 91% of the total variance. Factor 1 (F1) correlated positively with acids, C6-alcohols, esters, acetals and norisoprenoids, the latter two represented by 1-(1-ethoxyethoxy)-pentane and norisoprenoids by 3-oxo-α-ionol. All families whose concentration can be influenced by the presence of pollen are represented in this factor, being the esters the ones with the highest load (0.958). Factor 2 (F2) represented families of compounds not related to the influence of pollen. The decrease of some of these families could be related to ester formation. These could be considered as intermediate compounds or precursors of other compounds and therefore their concentration may fluctuate with the pollen dose. F2 could explain all the fluctuations in all families resulting from consumption of some compounds in order to form others. In some cases, it is observed families where at certain pollen doses, their concentrations are lower than the control. This behavior is possibly a reflection of the consumption of these compounds to form others. Factor 3 (F3) belongs to pollen response on the aromatic profile of wines. This factor involves volatile compound families displaying higher concentration increases with pollen presence, not correlated with doses.

F1 increased with the pollen dose, reaching higher levels for high doses (10 and 20 g/L). This suggests that the effect of ester formation is the main one, taking into account that it is one of the compound families with high participation in the aromatic profile of wines (10.86–22.34%). F2 positioned control and dose of 0.1 g/L with similar behaviors, while those doses that generate an increase mainly of higher alcohols, aldehydes and phenols advanced towards positive values. However, this factor is offset by the effect of the methanol. As previously observed, this compound exhibited fluctuations in its concentration, which means that F2 had no correlation with the pollen dose. Factors F3, together with F1, showed an increasing trend of both pollen-formed and pollen-produced compounds.

Therefore, it could be pointed out that pollen use in Tintilla de Rota grapes variety vinification favors to produce fruity and floral aromas compounds in wines.

#### 3.1.12. Odorant Activity Values (OAV) Analysis.

Odorant Activity Values (ΣOAV) of all volatile compounds involved in the aromatic profile of wines are shown in Table 3. Widely, there was an OAV increase between 30.7 and 63.6% in all pollen dosage wines without correlation. The most important odorant series in wines was fruity. Its values increased with the pollen addition (129.35–154.61), reaching maximum levels for 0.25 g/L, followed by 20 g/L and 5 g/L doses. Floral series reached maximum levels for doses between 0.25–1 g/L, while it began to decrease from the dose of 10 g/L. Spicy aromas were intensified an average 58% between 1–10 g/L of pollen dose. Fatty aroma series increased slightly in 5 g/L samples, increasing a 25% over ΣOAV_T_. On 1 g/L, herbal odorant series showed a slight increase, mainly caused by C6 alcohols, α-terpineol, *n*-propyl alcohol.

Despite the increase in “negative” odorants series (fatty and grassy), their influence on the total was very low (Table 3). It should be noted that higher bee pollen doses increased chemical odorant series and 1 g/L, 5 g/L and 10 g/L dosages increased earthy notes as well. In all cases, the highest load was observed in fruity, floral and spicy series, with values greater than 90% and much higher in all pollen dosage wines (<94%). Despite varietal character, increases in fruity, floral and spicy series would suggest that bee pollen promoted an increase of compounds enhancing wine aromatic profile quality. Low and intermediate pollen doses were those with the maximum levels of ΣOAV (fruits, flowers, and spices). Besides, the increase in fatty and herbaceous aromas, produced during winemaking with high doses, dropped the ratio Σ (fruits, flowers, spices)/Σ (fatty, herbaceous). These results proved that low doses of bee pollen promote fruit and floral aromas, enhancing varietal sensory quality in red wines.

### 3.2. Sensory Evaluation of the Resulting Wines.

**Figure 1:**
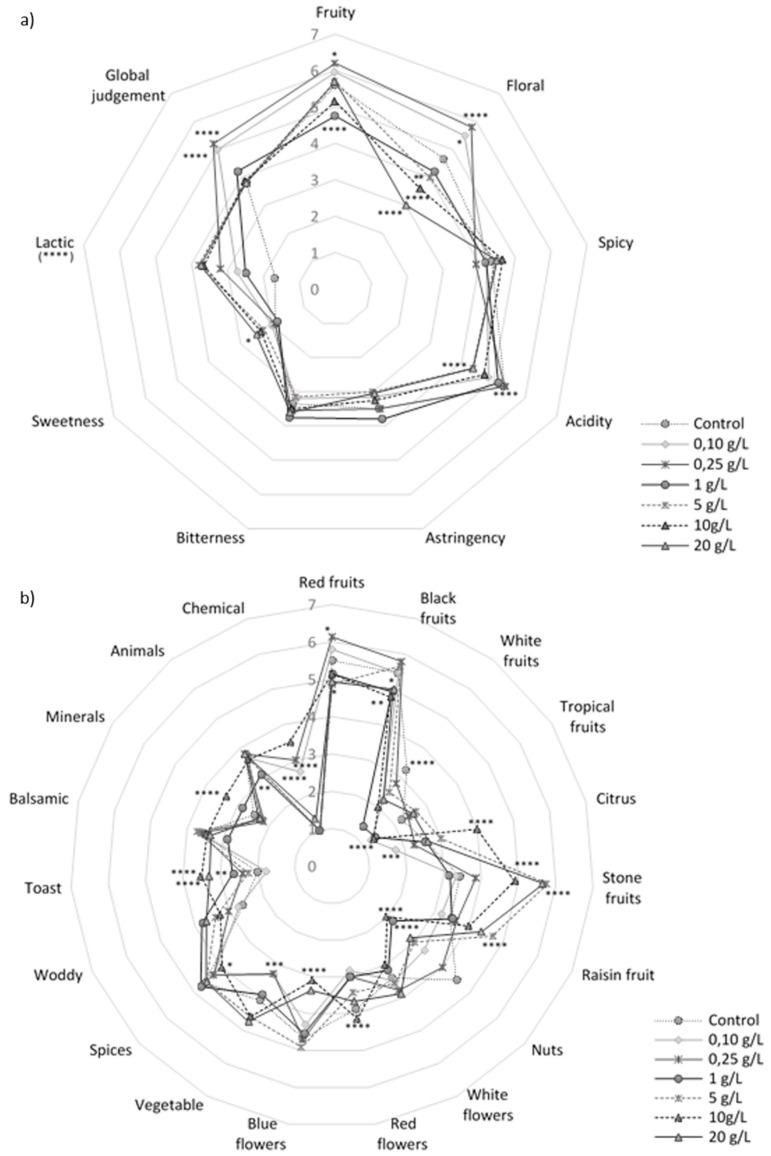
Tintilla de Rota red wines sensorial evaluation results of generic attributes (**a**), and specific olfactory attributes (**b**). * indicates level of significance for two-way ANOVA (BSD-test) (* *p* < 0.05, ** *p* < 0.01, *** *p* < 0.001 and **** *p* < 0.0001).

Generic and specific attributes average results with significant differences between the pollen wines and the control are shown in Figure 1a,b. Additional Table S1 show average numeric results of sensory analysis (generic and specific attributes). Each of the tasters was able to identify an average of 20 attributes. Tasters noted attributes related to fruit and floral aromas, and feelings of acidity and sweetness in mouth the most significant differences (ANOVA, *p* < 0.05) (Figure 1a). Low doses (0.1 and 0.25 g/L) were valued the best for fruit and floral attributes, being 0.25 g/L the best. Nevertheless, wines pungency increased with high bee pollen doses. An increase in spicy character would be related to a phenols and some esters increase, such as methyl vanilla, belonging to spicy odorant family.

For generic olfactory attributes, 0.25 g/L was the best evaluated followed by 0.10 g/L. Bitterness and astringency feelings, decreased from 5 g/L to 20 g/L compared to low doses and control, while sweetness feeling increased. For general taste attributes, 0.25 g/L and 1 g/L were the best scored wines. Thus, the 0.25 g/L bee pollen dose could be the best resulting dose in terms of general sensory (olfactory and gustatory) aspects. Wines with low doses of bee pollen improved their olfactory organoleptic qualities, increasing fruity and floral notes, highly appreciated by consumers in young red wines [46].

Compared with the control, all specific olfactory attributes (Figure 1b) showed significant differences excluding the white flower notes. Low doses were the best scored in red and black fruit attributes, being the best valued 0.25 g/L. Concerning citrus notes, a clear tendency was found with the pollen doses increase. Stone fruit and ripe or raisin fruit notes were significantly scored (ANOVA, *p* < 0.05) above low dose and control. This effect could be explained by oxidation notes produced from certain fatty acids [47], translated as ripe fruit or raisin notes by tasters.

Finally, it should be noted for red flower notes, low and intermediate pollen doses got the lowest values significantly compared to control. In contrast, the largest doses (10 and 20 g/L) had significantly lower values compared to the control. Also, all the large doses (5 g/L to 20 g/L) showed greater aromatic intensity in the vegetable notes. In addition, these doses showed a slight tendency to increase to spices, wood, toast, balsamic, minerals and animal’s notes, compared to the low, intermediate doses and control (Figure 1b).

## 4. Conclusions

In conclusion, the contribution of multiflora bee pollen to Tintilla de Rota red grape musts increases the concentration of total volatile compounds of final wines, especially the families of higher alcohols, esters, terpenes, phenols, thiols, and norisoprenoids families. Lower pollen doses (0.1 and 0.25 g/L) increases the total levels of the OAV and the series of aromas associated with the fruity and floral character of red wines, whereas high bee pollen doses enhance the chemical, fatty, and grassy aromatic series mainly. In addition, high and intermediate dose (1 g/L) produced an increase in the earthy notes in the aromas. Descriptive sensory analysis determines that low doses of pollen (0.1 and 0.25 g/L) obtain the highest scores in the overall assessment and sensory attributes responsible for fruity and floral aromas in red wines, highlighting red and black fruit attributes. However, the high doses diverted the sensory profile towards fleshy stone fruit, fruit with raisins, and more typical aromas of red wines with some evolution or aging.

## Figures and Tables

**Table 1:** Comparative effect of the addition of bee pollen (0.1, 0.25, 1, 5, 10 and 20 g/L) on concentrations of volatile compounds (µg/L) and control elaboration of Tintilla de Rota red wines.

Compounds	Tintilla de Rota Red Wines Pollen Doses																				
	Control			0.1 g/L			0.25 g/L			1 g/L			5 g/L			10 g/L			20 g/L		
*Higher alcohols*																					
2-Propanol	34,953.9	±	1276.1 ^a^	36,052.5	±	2210.1 ^a^	38,842.7	±	4907.9 ^a^	34,985.5	±	1788.9 ^a^	36,902.1	±	799.7 ^a^	34,768.3	±	1495 ^a^	33,171.9	±	815.5 ^a^
*n*-Propyl alcohol	27,691.4	±	2165.4 ^a^	28,078.3	±	3292.1 ^a,b^	27,692.6	±	1445 ^a^	33,332.2	±	3069.1 ^b,c^	29,588.4	±	720.7 ^a,c^	30,273.1	±	2148.2 ^a,c^	34,846.2	±	2039.4 ^c^
Isobutanol	31,385.1	±	1539.9 ^a,b^	31,248.3	±	1983.1 ^a^	39,121	±	683.8 ^b^	38,397.8	±	1745.5 ^a,b^	34,307.7	±	358.1 ^a,b^	36,460.3	±	1599.1 ^a,b^	50,569	±	1463.5 ^c^
Isoamyl alcohols	354,323.6	±	16,785.8 ^a,b^	350,155.6	±	21,853 ^a,b^	393,105.9	±	5534 ^a^	354,546.7	±	16,445 ^a,b^	316,247.7	±	3734.7 ^b^	347,556.9	±	15,377.3 ^a,b^	355,663.7	±	10,652.6 ^a,b^
Total	448,353.9	±	21,767.1	445,534.8	±	29,338.3	498,762.1	±	12,570.7	461,262.2	±	23,048.6	417,045.8	±	5613.1	449,058.5	±	20,619.7	474,250.8	±	14,971
% higher alcohols	77.53%			76.12%			77.82%			74.73%			70.49%			71.89%			67.65%		
*Methanol*	32,708.5	±	3833 ^a,b^	34,834.6	±	3461.2 ^a,c,d^	27,651.5	±	141.1 ^b^	40,294	±	5164 ^c,d,e^	40,450.6	±	1741.7 ^d,e^	43,161.7	±	5621 ^e^	33,131.9	±	4624.9 ^a,b^
Total	32,708.5	±	3833	34,834.6	±	3461.2	27,651.5	±	141.1	40,294	±	5164	40,450.6	±	1741.7	43,161.7	±	5621	33,131.9	±	4624.9
% methanol	5.66%			5.95%			4.31%			6.53%			6.84%			6.91%			4.73%		
*Acids*																					
Butanoic acid	30	±	0.2 ^a,b^	33	±	0.7 ^b^	34.2	±	0.8 ^c,d^	29.3	±	0.8 ^a^	32	±	2.5 ^b,c,d^	29.8	±	0.4 ^a,b^	29.4	±	0.7 ^a,b^
3-Methyl-butanoic acid	189	±	1.6 ^a^	190.4	±	2.7 ^a^	182.2	±	10.7 ^a^	188.8	±	13.5 ^a^	193.4	±	6.8 ^a^	189.9	±	14.1 ^a^	184.2	±	7.3 ^a^
Hexanoic acid	556.3	±	42.4 ^a^	651.6	±	5.8 ^b^	466.5	±	26.1 ^c,e,f^	390.4	±	4.5 ^d^	463.5	±	29.4 ^e,f^	477.2	±	3.3 ^f^	770.4	±	23 ^g^
Heptanoic acid	20.7	±	0.7 ^a^	24	±	0.6 ^a^	24.7	±	0.5 ^a^	25.6	±	0.7 ^a^	27.9	±	0.9 ^a^	74.8	±	0.9 ^b^	144.4	±	10.7 ^c^
2-Hexenoic acid	34	±	0.6 ^a^	39.4	±	0.6 ^b,c^	39.9	±	0.3 ^c^	44.6	±	1.6 ^d,e^	45.7	±	1.7 ^e^	53.7	±	4.5 ^f^	60	±	0.2 ^g^
Octanoic acid	624.4	±	19.8 ^a^	366.6	±	23.9 ^b,c^	443.7	±	30.9 ^c^	648.1	±	37.2 ^a^	1424.5	±	14 ^d,e^	1443.3	±	30.6 ^e^	1253.8	±	39.3 ^f^
Nonanoic acid	87.7	±	2.1 ^a^	76.4	±	3.4 ^a^	90.5	±	3.7 ^a^	116.2	±	1.4 ^a^	556.2	±	9.7 ^b^	182.6	±	0.4 ^c^	184.4	±	11.1 ^c^
*n*-Decanoic acid	0.19	±	0.01 ^a^	398.5	±	26.5 ^b,c,f^	412.4	±	1.3 ^c,f^	311.9	±	6.9 ^d^	866.4	±	46.2 ^e^	440.9	±	3.0 ^f^	450.3	±	39.6 ^f^
9-Decenoic acid	21.4	±	1.5 ^a^	154.3	±	12.4 ^b^	33.8	±	1.9 ^c,d^	39.1	±	0.8 ^d^	80.1	±	1.3 ^e,g^	52.7	±	2.7 ^f^	87.9	±	0.6 ^g^
Benzoic acid	84.2	±	5.9 ^a^	200.8	±	0.1 ^b^	108.7	±	1.4 ^a,c^	125	±	5.8 ^c^	328.8	±	29.8 ^d^	274	±	3.5 ^e^	371.3	±	12.2 ^f^
Benzeneacetic acid	40.8	±	0.4 ^a^	42.8	±	0.8 ^a,b,c^	41.9	±	1.1 ^a^	43.9	±	0.5 ^a,b,c^	46	±	0.7 ^b,c^	46.4	±	0.7 ^c^	49.5	±	2.2 ^c^
Total	1688.6	±	75.2	2177.7	±	77.5	1878.5	±	78.7	1962.8	±	73.8	4064.5	±	143.1	3265.3	±	64.1	3585.5	±	147
% acids	0.29%			0.37%			0.29%			0.32%			0.69%			0.52%			0.51%		
*C-6 alcohols*																					
1-Hexanol	493.9	±	9.5 ^a^	431.1	±	1.7 ^b,c,d^	436.6	±	19.2 ^c,d^	433	±	10.2 ^d^	434.2	±	20.2 ^e^	520.3	±	1.4 ^a^	539.5	±	53.1 ^a^
(E)-3-Hexen-1-ol	16.7	±	0.2 ^a^	29.1	±	0.5 ^b,c^	30.9	±	0.9 ^c,d^	33.1	±	0.8 ^d^	35.7	±	1.7 ^e^	36.1	±	0.6 ^e^	37.7	±	1.4 ^e^
(Z)-3-Hexen-1-ol	25.3	±	2.4 ^a^	30	±	1.4 ^a,d^	31.8	±	1.0 ^a^	91.2	±	0.4 ^b^	101.1	±	6.8 ^c^	6.2	±	0.3 ^d^	9.5	±	0.1 ^a,d^
Total	535.9	±	12.1	490.3	±	3.6	499.2	±	21	557.2	±	11.4	571	±	28.7	562.6	±	2.3	586.7	±	54.6
% C-6 alcohols	0.09%			0.08%			0.08%			0.09%			0.10%			0.09%			0.08%		
*Alcoholes*																					
3-Penten-2-ol	103.6	±	4.9 ^a^	45.1	±	2.4 ^b,c^	50.6	±	0.1 ^c^	63.6	±	4.7 ^d,e,f^	67.2	±	3.9 ^e,f^	70.9	±	0.1 ^f^	129.5	±	2.0 ^g^
1-Pentanol	1358.2	±	53.1 ^a^	1812.7	±	73.5 ^b,c,d,e^	1331.8	±	123.8 ^a^	1746.8	±	14.0 ^c,d,e^	1847.5	±	17.9 ^d,e^	1992.8	±	0.5 ^e^	2925.1	±	187.4 ^f^
3-Ethyl-2-pentanol	10.1	±	0.3 ^a^	12.7	±	0.7 ^b,c^	13.9	±	1.0 ^c^	16.6	±	0.1 ^d,e^	17.8	±	0.1 ^e^	20	±	0.2 ^f^	21.3	±	1.9 ^f^
4-Methyl-1-pentanol	13	±	0.7 ^a^	16	±	0.8 ^b,c,d^	17.3	±	0.6 ^c,d^	18.3	±	0.7 ^d,e^	20.5	±	0.4 ^e,f^	20.7	±	1.1 ^f^	25.8	±	1.7 ^g^
3-Methyl-1-pentanol	290.4	±	8.0 ^a^	278.7	±	2.9 ^a^	184.1	±	7.2 ^b^	320.1	±	20.5 ^c^	288.6	±	15.5 ^a^	273.6	±	4.3 ^a^	110.2	±	8.7 ^d^
3-Ethoxy-1-Propanol	99.3	±	0.7 ^a^	100.7	±	0.7 ^a^	112.4	±	2.8 ^b,c,e^	118.9	±	1.1 ^c,d,e,f^	125	±	0.8 ^d,e,f^	120	±	1.6 ^e,f^	125.9	±	0.6 ^f^
1-Octanol	20.8	±	1.0 ^a^	23	±	0.7 ^a,b^	24.7	±	0.4 ^b,c,d,e^	25.5	±	0.1 ^c,d,e,f^	25.9	±	0.1 ^d,e,f^	25.8	±	1.6 ^e,f^	27.6	±	0.7 ^f^
1-Nonanol	3.8	±	0.3 ^a^	2.4	±	0.2 ^b,c,d,e^	2.7	±	0.02 ^c,d,f^	2.4	±	0.2 ^d,e^	2.2	±	0.2 ^e^	2.8	±	0.2 ^f,g^	3.1	±	0.02 ^g^
Benzyl alcohol	84.5	±	7.1 ^a^	93.7	±	0.6 ^a^	133	±	10.6 ^a,b^	179.7	±	3.8 ^b^	603.9	±	58.2 ^c^	248.8	±	2.3 ^d^	247.7	±	18.8 ^d^
Phenylethyl Alcohol	23,942.5	±	1414.2 ^a^	23,512	±	235.9 ^a^	28,318.7	±	302.5 ^b,c^	24,110.5	±	1481.8 ^a^	27,830.4	±	2039.4 ^c^	22,209.8	±	331.3 ^a^	23,346.3	±	2021.2 ^a^
1 H-Indol-3-ethanol	862.1	±	25 ^a^	1313.5	±	10.6 ^b,c^	1452	±	118.6 ^c^	1823.4	±	2.6 ^d,e^	1866.8	±	64.1 ^e^	452	±	28.7 ^f^	551.7	±	6.5 ^f^
1-Butanol	31.2	±	1.6 ^a^	22.7	±	0.9 ^a,b^	15.3	±	0.9 ^b,e^	23.3	±	1.7 ^a,e^	114.3	±	5.8 ^c^	44.3	±	1.0 ^d^	29.7	±	0.8 ^a^
3-Metil-2-buten-1-ol	41.5	±	1.7 ^a^	25.8	±	1.2 ^b^	46.9	±	1.4 ^a,c^	49.3	±	4.3 ^c^	57.1	±	2.8 ^d^	65.5	±	4.1 ^e^	77.9	±	1.1 ^f^
Total	26,861	±	1518.6	27,259	±	331.2	31,703.5	±	570	28,498.3	±	1535.6	32,867.2	±	2209	25,547	±	377	27,622	±	2251.4
% Alcoholes	4.64%			4.66%			4.95%			4.62%			5.56%			4.09%			3.94%		
*Phenols*																					
2,6-di-terc-butil-4-ethylfenol	23.1	±	0.8 ^a^	29.1	±	0.5 ^b,c^	29.8	±	0.4 ^c^	20.1	±	0.1 ^d,e,f^	18.6	±	1.4 ^e,f^	20.8	±	0.9 ^a,f^	23.3	±	0.1 ^a^
4-Etilfenol	6.7	±	0.2 ^a^	8.2	±	0.4 ^b,e^	9.4	±	0.7 ^c,d,e^	10.1	±	0.1 ^d^	8.6	±	0.2 ^e^	4.9	±	0.5 ^f^	7.2	±	0.1 ^a^
4-Vinilguaiacol	33.4	±	1.1 ^a^	55.7	±	5.3 ^b^	76.6	±	3.7 ^c,d^	74.9	±	6.3 ^d^	23.1	±	0.8 ^e,f^	23	±	1.4 ^f^	41.5	±	0.3 ^g^
Acetovainillin	19.7	±	0.7 ^a^	32.7	±	0.8 ^b,d,f^	46.2	±	0.5 ^c^	32.6	±	0.7 ^d,f^	26.3	±	0.7 ^e^	20.5	±	1.4 ^a^	36.3	±	0.7 ^f^
Total	82.9	±	2.9	125.6	±	7	162	±	5.2	137.7	±	7.2	76.6	±	3.1	69.2	±	4.1	108.3	±	1.2
% Phenols	0.01%			0.02%			0.03%			0.02%			0.01%			0.01%			0.02%		
*Terpenes*																					
Linalool oxide	50.5	±	0.9 ^a^	74.2	±	1.8 ^b^	86.3	±	8.1 ^c,f^	59.1	±	0.8 ^d^	50.1	±	3.5 ^a^	38.2	±	0.6 ^e^	88.3	±	2.7 ^f^
Linalool	11.5	±	0.5 ^a^	12.9	±	0.8 ^a,b^	13.5	±	0.1 ^b^	16.7	±	0.3 ^c,e^	11.8	±	0.5 ^a^	18.4	±	0.8 ^d^	17	±	0.5 ^e,d^
α-Terpieol	9.7	±	0.2 ^a^	10.8	±	0.1 ^a^	15.3	±	0.1 ^b^	19.1	±	0.9 ^c,e^	23.2	±	1.4 ^d^	18.9	±	1.4 ^e^	34	±	2.4 ^f^
(R)-(+)-β-Citronellol	8.7	±	0.2 ^a^	9.7	±	0.2 ^a,b^	10.2	±	0.1 ^b^	13.8	±	0.9 ^c,e^	16.8	±	0.9 ^d,f^	12.7	±	0.8 ^e^	15.7	±	1.4 ^f^
2,6-dimetil-3,7-Octadiene-2,6-diol,	29	±	0.1 ^a^	32.8	±	0.4 ^b,e,f,g^	38.9	±	0.2 ^c,d,e^	41.2	±	1.5 ^d^	36.2	±	0.7 ^e,f^	33.3	±	0.6 ^f,g^	32.6	±	0.7 ^g^
8-Hydroxylinalool	49.4	±	2.1 ^a^	100.4	±	0.9 ^b,d,g^	202.4	±	0.3 ^c^	91.1	±	7.9 ^d^	387.1	±	8.9 ^e^	142.2	±	11.8 ^f^	130.3	±	9.8 ^f,g^
Total	158.8	±	4.1	240.8	±	4.3	366.7	±	8.9	240.9	±	12.4	525.2	±	15.8	263.7	±	16	318	±	17.4
% Terpenes	0.03%			0.04%			0.06%			0.04%			0.09%			0.04%			0.05%		
*Esters*																					
Ethyl acetate	60,290.1	±	5264.2 ^a^	65,998.8	±	4222.7 ^a,b^	68,955	±	3516.9 ^a,b^	75,224.6	±	6073.5 ^b,c^	85,400.4	±	1651.2 ^c,d^	94,930.8	±	4988.6 ^d^	151,421.2	±	10,126 ^e^
Ethyl butyrate	0.15	±	0.01 ^a^	27.2	±	0.7 ^b,g^	37.5	±	2.8 ^c^	55.3	±	1.8 ^d^	127.1	±	2.4 ^e^	16.7	±	0.1 ^f^	23	±	1.9 ^g^
Ethyl isovalerate	5.6	±	0.3 ^a^	14.5	±	0.2 ^b^	17.7	±	0.1 ^c^	20.9	±	0.8 ^d,g^	25.1	±	0.7 ^e^	27.6	±	0.1 ^f^	19.9	±	2.1 ^g^
Isoamyl acetate	29.4	±	0.9 ^a^	45.5	±	1.6 ^b,c^	50.5	±	0.3 ^c^	75.5	±	1.9 ^d^	180.1	±	3.8 ^e^	107.6	±	0.1 ^f^	126.9	±	1.4 ^g^
Ethyl hexanoate	60	±	0.1 ^a^	107.6	±	2.1 ^b,c,g^	105.1	±	0.7 ^c,g^	124.9	±	1.8 ^d,e,f^	118.9	±	0.3 ^e,h^	133.6	±	1.4 ^f^	115	±	4.3 ^g,h^
Hexyl acetate	26.3	±	0.01 ^a^	29.4	±	0.01 ^a,b^	30.3	±	0.01 ^b,c,d,e^	32.8	±	0.7 ^c,g^	28.3	±	1.4 ^a,e^	33.4	±	0.01 ^d,g^	42.6	±	2.1 ^f^
Ethyl 2-hydroxy-3-methyl butanoate	13.2	±	1.3 ^a^	15.8	±	0.9 ^a^	17.7	±	0.7 ^a^	29.6	±	0.8 ^b,e^	63.3	±	2.7 ^c^	37	±	1.4 ^d^	33.4	±	3.0 ^e^
Ethyl octanoate	204.5	±	6.1 ^a^	349.9	±	32.6 ^b,c,g^	372	±	0.6 ^c,g^	244.8	±	22.1 ^d,e,f^	261.2	±	26.5 ^e,f^	245.4	±	21.8 ^f^	346.7	±	3.1 ^g^
Ethyl nonanoate	10.9	±	0.9 ^a^	12.2	±	1.0 ^b,c,g^	11	±	0.6 ^a,g^	13	±	0.8 ^c^	16.3	±	0.6 ^d,e,f^	15.8	±	1.4 ^e^	17.1	±	0.5 ^f^
Ethyl 2-hydroxy-4-methylpentanoate	36.8	±	1.6 ^a^	36.2	±	3.1 ^a^	37.4	±	2.7 ^a^	82.6	±	0.7 ^b^	112.9	±	7.6 ^c^	39.3	±	1.9 ^a^	47.8	±	2.0 ^d^
Isoamyl lactate	43.3	±	1.8 ^a^	139	±	0.6 ^b,f^	229.3	±	3.1 ^c^	101.2	±	1.0 ^d^	255.5	±	7.1 ^e^	146.6	±	2.1 ^f^	127.8	±	0.4 ^f^
Ethyl decanoate	112.1	±	7.1 ^a^	122.4	±	0.3 ^a^	154.8	±	3.6 ^b^	111.8	±	5.3 ^a^	270.2	±	7.5 ^c,d^	127.1	±	1.6 ^a^	273.9	±	5.8 ^d^
Diethyl succinate	509.3	±	12.2 ^a^	681.5	±	2.5 ^b,d,e^	950.9	±	48 ^c,f^	636.5	±	51.2 ^d,e^	633.3	±	44.2 ^e^	907.9	±	43.2 ^f^	1310.2	±	125.7 ^g^
Ethyl 9-decenoate	59.7	±	0.8 ^a,c^	63.9	±	2.3 ^a,b^	66.1	±	0.8 ^b,d^	64.3	±	1.3 ^a,b,d^	59.4	±	0.7 ^a^	56.5	±	1.3 ^c^	62.8	±	0.8 ^a,b,d^
Ethyl phenylacetate	1.27	±	0.01 ^a^	2.23	±	0.01 ^b,c,d,f,g^	2.27	±	0.03 ^c,d,f,g^	2.39	±	0.07 ^d,e,f^	2.55	±	0.04 ^e^	2.27	±	0.08 ^f,g^	2.2	±	0.08 ^g^
Phenethyl acetate	33.8	±	0.1 ^a^	97.4	±	6.8 ^b,e,d,f^	104.5	±	4.1 ^c,d^	94.9	±	2.6 ^d,f^	279.1	±	5.1 ^e^	80.9	±	2.6 ^f^	251	±	3.1 ^g^
Diethyl malate	24.4	±	0.7 ^a^	32.9	±	0.9 ^b,c,d^	34.9	±	0.9 ^c,d,e^	36	±	0.9 ^d,e^	37	±	0.6 ^e^	40.6	±	0.7 ^f^	45.1	±	0.9 ^g^
Methyl vanillate	242.7	±	0.5 ^a^	287.4	±	5.2 ^a^	610.1	±	8.2 ^b,c^	550.3	±	45.8 ^c,e,f^	1541.4	±	18.6 ^d^	454	±	44.7 ^e,f^	470.4	±	62.1 ^f^
Ethyl lactate	121.4	±	1.5 ^a^	173.8	±	5.3 ^b,c^	155.9	±	4.3 ^c^	204.6	±	21.0 ^d,e^	199	±	3.2 ^e^	271.1	±	25.7 ^f^	256	±	4.0 ^f^
Butanoic acid 3-hydroxy ethyl ester	51.8	±	2.5 ^a^	63	±	1.3 ^a,b^	57	±	5.2 ^a^	76	±	3.5 ^b,d^	231.3	±	3.8 ^c^	82.7	±	1.3 ^d^	105.1	±	8.0 ^e^
Ethyl (Z)-4-decenoate	60.9	±	1.2 ^a^	165.9	±	3.7 ^b,d^	203.7	±	17.7 ^c^	191.3	±	6.1 ^d,c^	391.5	±	6.4 ^e^	31.9	±	1.6 ^a^	57.4	±	0.6 ^a^
Ethyl dodecanoate	68.2	±	2.6 ^a^	35.6	±	2.0 ^b^	143.5	±	14.1 ^c,f^	172	±	2.8 ^d^	303.9	±	8.1 ^e^	121.1	±	1.0 ^f^	91.8	±	0.3 ^g^
Methyl tetradecanoate	34.8	±	1.5 ^a^	66.4	±	7.2 ^b,c,d,f^	88.5	±	6.6 ^c,d,f^	72.8	±	3.8 ^d,f^	297.9	±	18.1 ^e^	80.5	±	0.8 ^f^	87.5	±	1.9 ^f^
Succinoic acid 2-hydroxy-3-methyl diethyl ester	93.3	±	8.1 ^a^	110.3	±	10.4 ^a,b,e^	126.9	±	3.4 ^b,c,e,f^	144.2	±	4.8 ^c,e,f^	450.8	±	36.4 ^d^	131.8	±	1.5 ^e^	147.2	±	2.3 ^f^
Methyl hexadecanoate	81.4	±	4.6 ^a^	100.9	±	5.2 ^b,e^	147.6	±	0.7 ^c^	219.3	±	10.1 ^d,g^	106.3	±	2.1 ^e^	186.4	±	4.3 ^f^	227.6	±	5.2 ^g^
Hexadecanoic acid, ethyl ester	310.8	±	7.4 ^a,f,g^	420.9	±	33.5 ^b,d^	732.6	±	20.6 ^c,e^	435.7	±	10.9 ^d^	719.8	±	61.2 ^e^	300.3	±	2.5 ^f^	356.1	±	14.5 ^g^
Propanoic acid 2-methyl-propyl ester	84.7	±	0.8 ^a^	116.1	±	11.1 ^b^	160	±	2.4 ^c,d,f^	154.5	±	4.5 ^d,f^	181.7	±	0.1 ^e^	147.7	±	8.1 ^f^	155.2	±	9.2 ^f^
Ethyl 8-nonenoato	205.6	±	3.5 ^a^	216.4	±	3.1 ^a,b^	235.9	±	0.8 ^b,f^	209.4	±	2.2 ^a,f^	293.9	±	6.5 ^c,d^	313	±	17.6 ^d^	363.5	±	20.5 ^e^
Total	62,816.5	±	5332.3	69,533.2	±	4366.3	73,838.8	±	3669.9	79,381.1	±	6282.9	92,587.9	±	1927.1	99,069.5	±	5177.4	156,584.3	±	10,411.7
% esters	10.86%			11.88%			11.52%			12.86%			15.65%			15.86%			22.34%		
*Aldehydes*																					
Acetaldehyde	4890.4	±	279.7 ^a,b^	4810.7	±	204.3 ^a^	5520.7	±	263.1 ^b^	4482.1	±	472.6 ^a^	2887.5	±	294.1 ^c,d^	3292.9	±	322.7 ^d^	4422.9	±	417.9 ^a^
Benzeneacetaldehyde	66.6	±	1.9 ^a^	74.3	±	5.7 ^a,e^	131.9	±	10.4 ^b,c,d^	145.3	±	11.7 ^c^	119.8	±	7.0 ^d^	80.5	±	3.8 ^a,e^	85.1	±	2.6 ^e^
Nonanal	10.5	±	0.6 ^a,e^	13.4	±	0.4 ^b,d^	16.2	±	1.0 ^c^	14.2	±	0.5 ^d^	10.7	±	0.6 ^a^	9.9	±	0.1 ^a,e^	8.6	±	0.7 ^e^
3-methyl-butanal	15.1	±	0.5 ^a^	28.5	±	1.5 ^b,c^	30.1	±	0.5 ^c,d^	35.1	±	2.6 ^d^	48.8	±	2.6 ^e^	10	±	0.3 ^a^	15.4	±	0.4 ^a^
Total	4982.6	±	282.7	4926.8	±	212	5698.9	±	275	4676.8	±	487.4	3066.8	±	304.4	3393.3	±	326.9	4532.1	±	421.6
% Aldehydes	0.86%			0.84%			0.89%			0.76%			0.52%			0.54%			0.65%		
*Thiols*																					
3-(methylthio)-1-Propanol	21.4	±	1.8 ^a^	49.3	±	3.8 ^b^	148.9	±	7.5 ^c^	95.7	±	3.7 ^d,f^	198.5	±	3.2 ^e^	88.1	±	8.6 ^f^	111.7	±	2.4 ^g^
Total	21.4	±	1.8	49.3	±	3.8	148.9	±	7.5	95.7	±	3.7	198.5	±	3.2	88.1	±	8.6	111.7	±	2.4
% thiols	0.004%			0.01%			0.02%			0.02%			0.03%			0.01%			0.02%		
*Acetals*																					
1-(1-etoxietoxi)-pentano	2.1	±	0.1 ^a^	2	±	0.1 ^a^	1.8	±	0.1 ^a^	3.2	±	0.1 ^b^	4.4	±	0.2 ^c^	5.4	±	0.1 ^d^	5.7	±	0.2 ^e^
Total	2.1	±	0	2	±	0	1.8	±	0.1	3.2	±	0	4.4	±	0.2	5.4	±	0.1	5.7	±	0.2
% acetals	0.0004%			0.0003%			0.0003%			0.0010%			0.0010%			0.0010%			0.0010%		
*Norisoprenoids*																					
3-Oxo-α-ionol	7.6	±	0.1 ^a^	13.3	±	0.4 ^b,c,d,e^	13.1	±	0.1 ^c,d,e^	14.1	±	0.2 ^d,e^	14.7	±	0.1 ^e^	42.8	±	0.6 ^f^	44.9	±	1.0 ^f^
Total	7.6	±	0.1	13.3	±	0.4	13.1	±	0.1	14.1	±	0.2	14.7	±	0.1	42.8	±	0.6	44.9	±	1
% Norisoprenoids	0.001%			0.002%			0.002%			0.002%			0.002%			0.010%			0.010%		
*Lactones*																					
Dihydro-5-pentyl-2-(3 H)-Furanona	59.6	±	0.7 ^a^	55.8	±	4.8 ^a^	107.2	±	2.8 ^b,d^	78.8	±	0.5 ^c^	106.8	±	0.3 ^d^	66.8	±	0.2 ^a^	120.6	±	8.4 ^e^
2,3-dihydro-benzofuran	50.6	±	1.1 ^a^	50	±	4.0 ^a^	50.6	±	1.1 ^a^	42.3	±	0.8 ^b^	49.5	±	0.8 ^a^	48.8	±	1.5 ^a^	50.1	±	1.6 ^a^
Total	110.2	±	1.7	105.8	±	8.8	157.8	±	4	121.1	±	1.4	156.2	±	1.1	115.6	±	1.7	170.7	±	10
% lactones	0.02%			0.02%			0.02%			0.02%			0.03%			0.02%			0.02%		

Different superscript letters indicate that there are significant differences between the samples (*p* < 0.05, ANOVA, BSD test).

**Table 2:** Loadings of the main components of the volatile compounds in Tintilla de Rota dosed with bee pollen and control wines.

	F1	F2	F3
Higher alcohols	0.179	0.936	−0.059
Methanol	0.193	−0.862	−0.082
Acids	0.648	−0.533	0.492
C-6 alcohols	0.744	−0.449	0.151
Alcohols	−0.282	0.102	0.948
Phenols	−0.284	0.831	0.152
Terpenes and derivatives	0.129	−0.196	0.961
Esters	0.958	0.056	0.164
Aldehydes	−0.355	0.895	−0.242
Thiols	0.190	−0.080	0.955
Acetals	0.895	−0.420	0.121
Norisoprenoids	0.945	−0.079	−0.140
Lactones	0.513	0.336	0.769
Explained variance (%)	32.75	30.37	28.80

Rotated component matrix loadings of Principal Components Analysis of volatile compounds in Tintilla de Rota red wines, using varimax with Kaiser normalization.

**Table 3:** Odor activity values summary (ΣOAV) grouped by odorant series.

Odorant Series	Bee Pollen Doses in Tintilla de ROTA Red Wines						
	Control	0.1 g/L	0.25 g/L	1 g/L	5 g/L	10 g/L	20 g/L
Fruity	93.03	142.21	154.61	134.68	146.87	129.35	151.40
Floral	25.25	31.01	45.26	43.24	40.41	26.58	36.55
Fatty	8.50	8.67	8.15	8.46	10.89	10.47	10.69
Grassy	1.15	1.15	1.17	1.45	1.42	1.26	1.45
Dry fruit	0.05	0.05	0.06	0.05	0.03	0.03	0.04
Earthy, mushrooms	0.60	0.61	0.52	0.74	0.78	0.64	0.33
Chemical	0.10	0.22	0.13	0.15	0.35	0.29	0.39
Spicy	0.94	1.52	2.17	2.09	1.12	0.75	1.23
Phenolic	0.02	0.02	0.02	0.02	0.02	0.01	0.02
∑OAV_T_	129.62	185.45	212.09	190.86	201.89	169.38	202.10

Odor Activity Values (OAV) sum of the main odorants found in Tintilla de Rota red wines produced (Control and different bee pollen doses addition (from 0.1 to 20 g/L)). ΣOAVT means the total sum of ΣOAV.

## Supplementary Materials

The following are available online at https://www.mdpi.com/2304-8158/9/8/981/s1. Table S1. Generic and specific olfatory attributes results of Tintilla de Rota wines sensory analysis.
