# Supplementary material for: Bee Pollen Role in Red Winemaking: Volatile Compounds and Sensory Characteristics of Tintilla de Rota Warm Climate Red Wines
Source: Foods. 2020 Jul 23;9(8):981. doi: 10.3390/foods9080981 (PMC7466273; doi:10.3390/foods9080981)
Supplement: Supplementary file 1 [file foods-09-00981-s001.pdf]

Supplemental Table 1.

Table S1. Generic and specific olfatory attributes results of Tintilla de Rota wines sensory analysis.

Sensorial Analysis - Generic Attibutes							
	**Control**	**0.10 g/L**	**0.25 g/L**	**1 g/L**	**5 g/L**	**10g/L**	**20 g/L**
Fruity	5.6	5.95	6.2	4.75	5.7	5.15	5.7
Floral	4.7	5.5	5.8	4.2	4.0	3.6	3.0
Spicy	4.4	4.2	3.9	4.2	4.5	4.65	4.5
Acidity	5.4	4.85	5.4	5.15	4.4	4.7	4.4
Astringency	3.5	3.15	3.5	3.8	3.0	3.25	3.1
Bitterness	3.4	3.2	3.6	3.75	3.2	3.5	3.6
Sweetness	1.9	2	1.9	1.8	2.3	2.35	2.5
Lactic	1.7	2.8	3.2	2.5	3.8	3.7	3.8
Global judgement	3.8	5.0	5.2	4.2	3.8	3.9	3.8

Sensorial Analysis - Specific Olfatory Attributes							
	**Control**	**0.10 g/L**	**0.25 g/L**	**1 g/L**	**5 g/L**	**10g/L**	**20 g/L**
Red fruits	5.5	5.8	6.1	5.1	4.9	5.1	4.9
Black fruits	5.5	5.5	5.8	4.9	5.7	4.8	5.0
White fruits	3.3	2.2	2.8	1.3	2.5	2.0	2.3
Tropical fruits	2.3	1.3	2.4	1.4	2.7	1.3	2.6
Citrus	2.6	1.8	2.3	2.6	3.0	4.0	2.7
Fruits with bone	3.4	3.3	3.9	3.2	5.8	4.9	5.7
Passion fruit	3.5	3.2	3.6	3.5	4.7	4.0	4.4
Nuts	4.5	3.3	4.0	2.2	3.0	2.0	2.8
White flowers	3.4	3.5	3.8	3.2	3.6	3.0	3.9
Red flowers	3.9	2.8	3.0	3.0	3.4	4.1	3.7
Blue flowers	4.7	4.3	4.7	4.5	4.9	3.1	3.4
Vegetable	4.1	3.3	3.3	3.9	4.6	4.6	4.7
Spices	4.6	4.3	4.3	4.8	4.6	4.0	4.6
Wood	2.6	2.7	3.0	3.8	3.4	3.3	3.7
Torrefacto	2.0	1.8	2.4	2.7	2.3	3.5	3.3
Balsamic	3.6	3.7	3.6	2.9	3.8	3.4	3.3
Minerals	2.5	2.3	2.2	2.8	2.3	3.4	2.3
Animals	3.8	3.7	3.8	3.1	3.7	3.6	3.8
Microbiological	1.0	2.7	3.0	1.0	1.0	3.5	1.3
